# Global research trends and frontiers in patent foramen ovale closure: a comprehensive bibliometric analysis (2004–2024)

**DOI:** 10.3389/fneur.2025.1618910

**Published:** 2025-07-22

**Authors:** Jie Liu, Yehong Liu, Ying Sheng, Jiangping Ye, Rikang Yuan, Xiao Wang, Gangjun Zong

**Affiliations:** Department of Cardiology, No. 904 Hospital of the PLA Joint Logistics Support Force, Wuxi, China

**Keywords:** patent foramen ovale closure, bibliometric analysis, cryptogenic stroke, atrial fibrillation, percutaneous intervention, global research trends

## Abstract

**Background:**

Patent foramen ovale (PFO), present in 20–30% of the population, was once considered benign but is now recognized as a contributor to cryptogenic stroke and other clinical syndromes. Recent randomized trials and updated guidelines have established PFO closure as an effective intervention, leading to a surge in research. This study uses bibliometric analysis to evaluate global research trends, collaborations, and emerging hotspots in PFO closure.

**Methods:**

We analyzed 927 English-language articles (2004–2024) from the Web of Science Core Collection using bibliometric tools (VOSviewer, CiteSpace, Bibliometrix R, online bibliometric analysis platforms). We systematically examined publication trends, contributions by countries and institutions, author networks, journal influence, and keyword clusters.

**Results:**

Annual publications increased significantly after 2017, coinciding with pivotal trial results. The United States (34.6%), Italy (16.8%), and Germany (11.5%) led in research output. Key institutions (e.g., University of Bern) and prominent authors (e.g., Meier Bernhard) played central roles. Four major research clusters were identified: mechanisms of paradoxical embolism, diagnostic imaging (e.g., transesophageal echocardiography), closure techniques (e.g., Amplatzer devices), and clinical outcomes. Burst detection revealed evolving priorities, including post-closure atrial fibrillation and improved patient selection (e.g., RoPE score).

**Conclusion:**

Research on PFO closure has progressed from pathophysiological understanding to evidence-based clinical intervention, driven by landmark trials and multidisciplinary collaboration. Future directions include optimizing patient selection, managing post-procedural complications, and expanding indications (e.g., migraine). This analysis offers a roadmap for advancing stroke prevention strategies related to PFO.

## 1 Introduction

Patent foramen ovale (PFO), present in approximately 20–30% of the population (1), was once considered a benign condition and thus received limited attention. As early as 1877, the German pathologist Cohnheim proposed a link between PFO and stroke (2). In recent years, a series of large randomized controlled trials (RCTs) have led to the recognition of PFO closure as a treatment for PFO-related strokes, resulting in its inclusion in several clinical guidelines (3). Advances in clinical research have significantly deepened our understanding of PFO-related conditions, including their disease spectrum, pathogenesis, and risk assessment. Consequently, the number of PFO closures performed has increased rapidly. Beyond the established research areas of PFO closure in stroke and migraine treatment, studies have also reported associations between PFO and conditions such as epilepsy, unexplained syncope, and others.

Bibliometrics is a method of literature analysis (4, 5) used to evaluate the research productivity of countries, institutions, and authors, and to identify development trends and emerging hotspots in specific scientific fields (6). Visualization tools such as CiteSpace (6.3. R1 Advanced), VOSviewer (1.6.20), Bibliometrix R (4.4.1), and the Online Analysis Platform for Bibliometrics are widely applied in bibliometric studies. CiteSpace is mainly used to generate keyword-based visual maps, helping users trace the developmental trajectory of a discipline and identify key turning points and significant trends. VOSviewer facilitates visual analysis of fundamental metrics such as countries, institutions, authors, and journals. Bibliometrix R supports the analysis of collaborative networks among countries or regions, evaluates the influence of core journals, and maps keyword themes. This study uses bibliometric methods to analyze the body of literature on PFO, aiming to provide insights into current research trends and the evolving landscape of this field.

## 2 Materials and methods

### 2.1 Data retrieval and collection

The retrieval was conducted on September 19, 2024. A keyword search with synonym expansion was performed using the Science Citation Index Expanded of the Web of Science Core Collection (WOSCC), covering the period from January 1, 2004, to September 19, 2024. To enhance accuracy in the bibliometric results, the search was conducted using a combination of title (TI), abstract (AB), and author keywords (AK). The search strategy was as follows: TI = (“patent foramen ovale closure” OR “PFO closure”) OR AB = (“patent foramen ovale closure” OR “PFO closure”) OR AK = (“patent foramen ovale closure” OR “PFO closure”). A total of 1,456 documents were retrieved. After excluding non-English publications and document types such as case reports, meeting abstracts, editorial materials, and other non-research articles, a final set of 927 English-language papers was obtained. This included 763 original research articles and 164 reviews. These articles were exported in plain text format and saved as “download_***.txt.”

### 2.2 Data analysis

Web of Science citation reports were used to calculate both annual and cumulative publication volumes. Bar charts illustrating trends in PFO closure research over the past 21 years were generated using Microsoft Excel (Office 2021).

A chart showing annual publication counts for the top 10 countries by research output was created using the online bibliometric analysis platform.^1^

International collaboration among countries and regions was visualized using Bibliometrix R (4.4.1). Bradford’s Law was applied to identify core journals in the PFO closure field. Additionally, a thematic map was developed through keyword analysis.

Network visualization analyses of countries/regions, authors, and institutions were performed using VOSviewer (1.6.20). Both network and overlay visualizations were conducted for keywords. In these maps, the shorter the distance between nodes, the greater the similarity between the topics they represent (7).

CiteSpace (6.1. R1 Advanced) (8) was used to identify emerging research hotspots and monitor dynamic trends through burst detection of keywords and references. A timeline map of keyword clusters derived from co-cited literature was also generated using CiteSpace to enable in-depth temporal analysis.

## 3 Results

### 3.1 Main information

**Figure 1:**
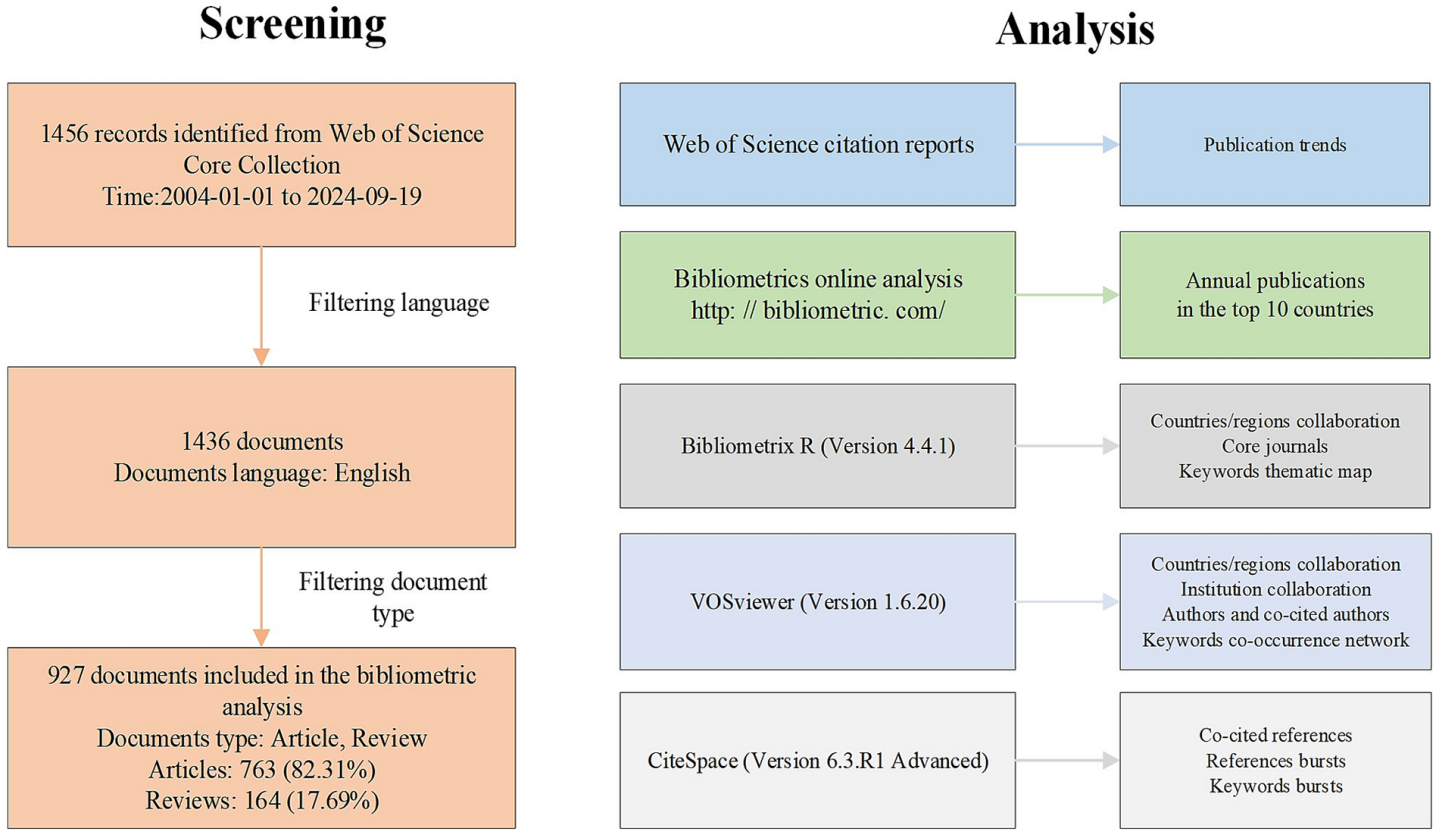
Flow chart of the literature search.

A total of 927 PFO closure-related research papers published between January 1, 2004, and September 19, 2024, were retrieved from the WOSCC database, comprising 763 original articles and 164 reviews. Figure 1 presents the flowchart for literature screening and bibliometric analysis.

### 3.2 Global publication trend

**Figure 2:**
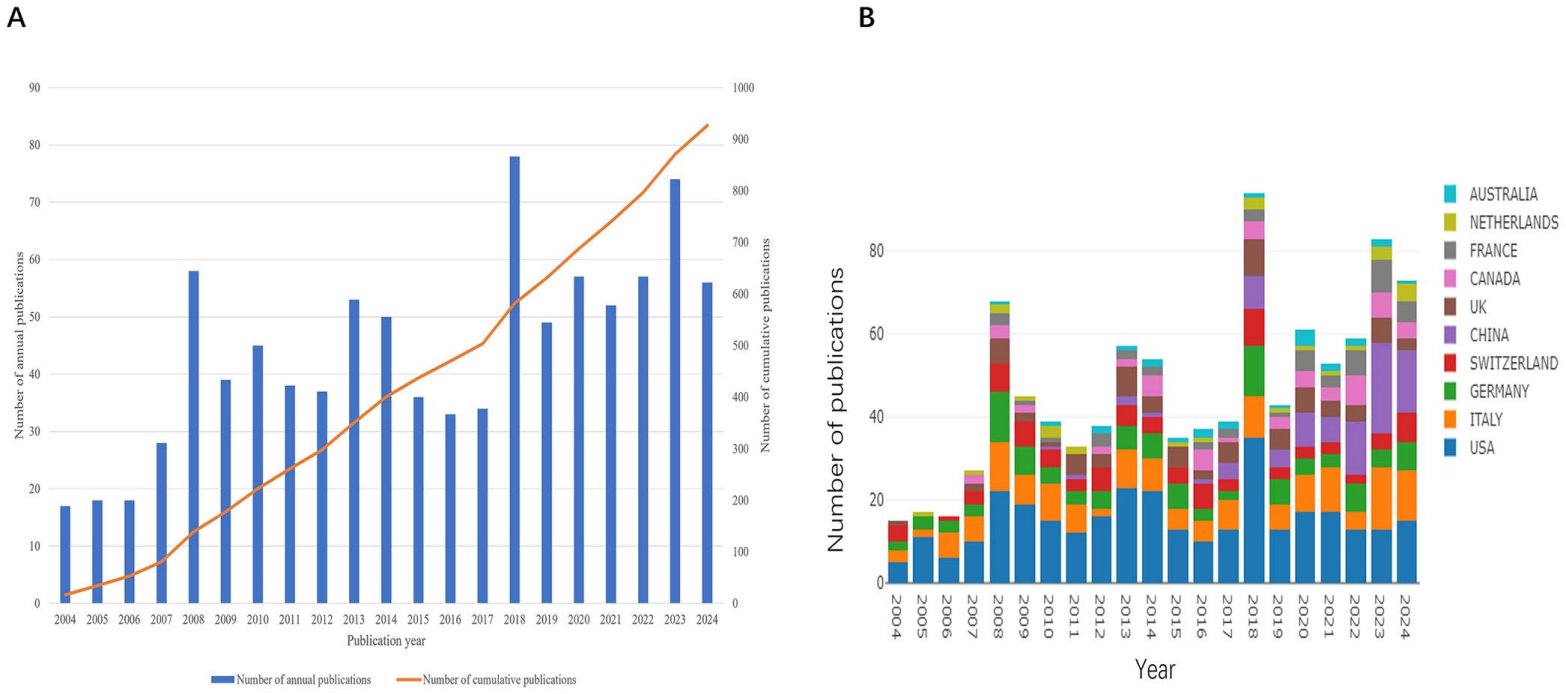
Global trends in publications on PFO. **(A)** Number of publications per year and the cumulative number. **(B)** Dynamics of publications in the top 10 countries between 2004 and 2024.

The number of publications increased steadily from 17 in 2004 to 74 in 2023 (Figure 2A). From 2004 to 2017, the growth was gradual, with annual outputs rarely exceeding 50. A notable rise occurred between 2018 and 2024, despite a slight dip in 2019 (<50 publications). In the past 7 years alone, 423 papers were published, averaging approximately 60 per year, reflecting a growing research interest in this field.

The top 10 countries/regions by publication volume are shown in Figure 2B. Over the 21-year period, the leading contributors were the United States (n = 321, 34.62%), Italy (n = 156, 16.82%), and Germany (n = 107, 11.54%). Although Chinese researchers entered the field later, their output surged after 2017. Over the past 3 years, China has surpassed all other countries in annual publication volume, highlighting its growing focus on PFO closure research.

### 3.3 Countries/regions analysis

**Figure 3:**
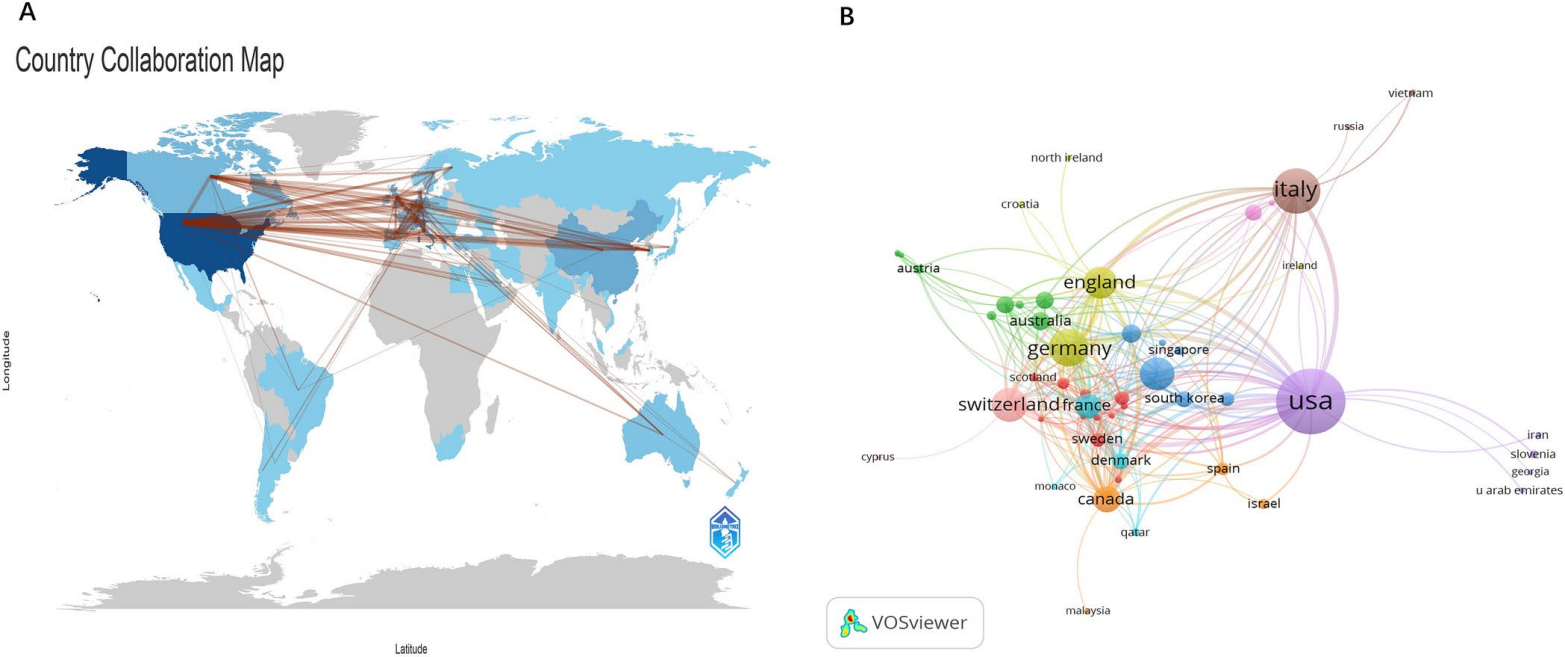
Graphical representation of collaboration among countries or regions. **(A)** Country scientific production map. Blue intensity is proportional to the number of publications (dark blue = high productivity; gray = no documents). The nationality of each author in a document was considered. **(B)** Network map of collaborative networks countries of PFO closure.

A total of 56 countries have contributed to PFO closure research. International collaboration was analyzed using Bibliometrix R (Figure 3A). The United States leads in overall research output and collaborates most frequently with Germany (30 collaborations) and Switzerland (21 collaborations). The collaboration network in Figure 3B illustrates publication volume (represented by circle size) and partnership strength (represented by line thickness). Total Link Strength (TLS), which reflects the intensity of co-authorship, identifies the top five countries as follows: the United States (TLS = 213), Germany (TLS = 148), England (TLS = 140), Switzerland (TLS = 129), and Canada (TLS = 110). In terms of publication count, the top contributing countries are the United States, Italy, Germany, Switzerland, and China. The network map demonstrates active global cooperation, particularly between China and countries such as the United States, Germany, the UK, Canada, and Switzerland, as well as strong ties between the United States and several European nations.

Table 1 summarizes the top 10 countries/regions by number of publications, total citations, and h-index. The United States leads with over one-third of global output (accounting for 85.33% of publications among the top 10), the highest number of citations (11,178), and the highest h-index (51). Switzerland ranks second in citations (3,780) and has an h-index of 32.

**Table 1:** Top 10 countries or regions that contributed publications on PFO closure.

Rank	Country	Documents	Citations	Average citation/Publication	H-index
1	USA	321	11,178	34.82	51
2	Italy	156	2033	13.03	25
3	Germany	107	3,037	28.38	25
4	Switzerland	88	3,780	42.95	32
5	China	83	493	5.94	12
6	England	79	2,775	35.13	24
7	Canada	54	2,819	52.2	23
8	France	48	1,462	30.46	15
9	Netherlands	26	330	12.69	11
10	Australia	25	896	35.84	10

### 3.4 Institution analysis

**Figure 4:**
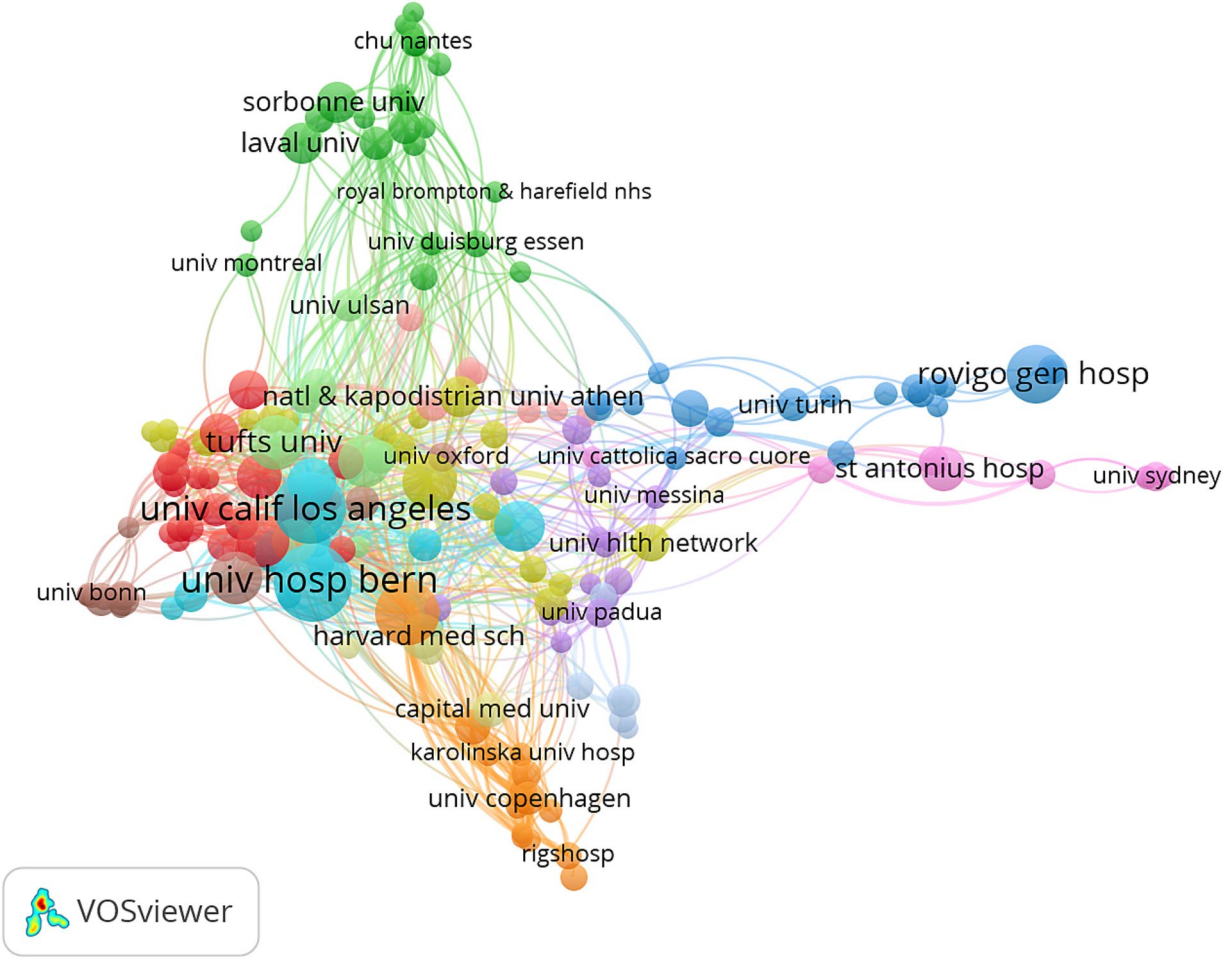
Network visualization of collaborative efforts between institutions.

A total of 1,393 institutions have contributed to research in this field. Among them, the top 10 institutions based on TLS collectively produced 230 papers. Of these, seven are based in the United States, two in Switzerland, and one in Canada (Figure 4A). Institutions in the United States and Switzerland are particularly prominent in terms of publication volume, with the University of Bern, the University of California, Los Angeles, and the University of Pennsylvania leading the field. To further analyze collaboration patterns, 209 institutions were selected for visualization, applying a threshold of at least three publications. A collaboration network was constructed based on the number of publications per institution and their inter-institutional relationships.

### 3.5 Authors, co-cited authors, co-cited references, and references burst analysis

**Figure 5:**
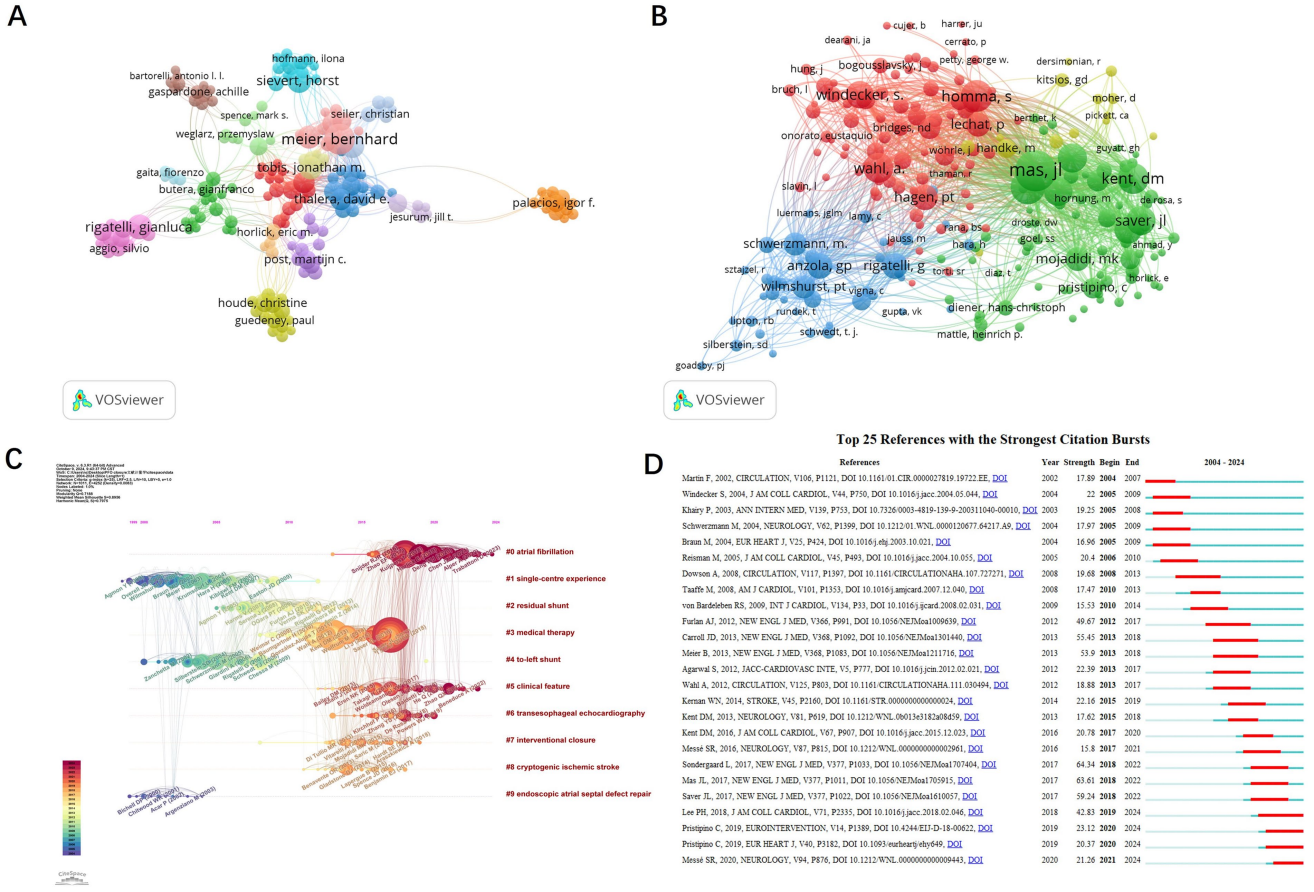
Analysis of authors and references of PFO closure-related publications. Network visualization of authors **(A)** and co-cited authors **(B)** generated by VOSviewer. Timeline view of the co-cited references **(C)** and the top 25 references with the strongest citation bursts generated using CiteSpace **(D).**

A total of 4,209 authors have contributed to PFO closure research. The co-authorship network (Figure 5A) illustrates collaborative relationships, where node size represents publication output and line thickness reflects the strength of collaboration. The five leading authors by TLS are: Meier Bernhard, Mattle Heinrich P., Rigatelli Gianluca, Windecker Stephan, and Kasner Scott E. These researchers have significantly advanced the field through extensive international collaboration. Co-citation analysis of 7,156 authors (Figure 5B) identified six authors who were co-cited more than 300 times: Mas JL (598 citations), Meier B (410), Homma S (345), and Kent DM (334). The network shows strong links between key figures such as Mas JL, Kent DM, and Homma S., emphasizing their foundational contributions to the field. Timeline clustering of co-cited references (Figure 5C) revealed evolving research trends. Earlier studies focused on single-center experiences and right-to-left shunts, whereas more recent work highlights atrial fibrillation and clinical characteristics. Sudden detection refers to identifying a sharp rise in citation frequency of a specific topic, article, author, or journal over a defined time period (9). Citation burst analysis identified 25 high-impact publications (Figure 5D). The strongest burst (64.34) was for the 2017 New England Journal of Medicine (NEJM) study by Søndergaard et al., which demonstrated a reduced risk of recurrent stroke with PFO closure plus antiplatelet therapy compared to antiplatelet therapy alone, although it also reported higher rates of device-related complications and atrial fibrillation. Four recent citation bursts indicate emerging directions in PFO closure research.

The most frequently cited articles in the field were also analyzed. Table 2 lists the top 10 most cited publications, each with more than 150 citations. The top three were all published in the NEJM. In 2012, Furlan et al. reported that device closure did not provide greater benefit than medical therapy for patients with cryptogenic stroke or transient ischemic attack (TIA) associated with PFO. In 2013, Meier et al. concluded that PFO closure for secondary prevention of cryptogenic embolism did not significantly reduce the risk of recurrence or mortality compared with medical therapy. In 2017, Mas et al. showed that PFO closure combined with antiplatelet therapy reduced the recurrence of cryptogenic stroke compared to antiplatelet therapy alone. However, several studies have also noted an increased risk of atrial fibrillation following PFO closure.

**Table 2:** Top 10 most cited research papers.

Rank	Title	Author	Year	journal	Citations	Total link strength
1	Closure or medical therapy for cryptogenic stroke with patent foramen ovale	Anthony J. Furlan	2012	New Engl J Med	267	10,023
2	Percutaneous closure of patent foramen ovale in cryptogenic embolism	Bernhard Meier	2013	New Engl J Med	258	9,368
3	Patent Foramen Ovale Closure or Anticoagulation vs. Antiplatelets after Stroke	Jean-Louis Mas	2017	New Engl J Med	229	9,187
4	Patent Foramen Ovale Closure or Antiplatelet Therapy for Cryptogenic Stroke	Lars Sondergaard	2017	New Engl J Med	246	9,017
5	Incidence and size of patent foramen ovale during the first 10 decades of life: an autopsy study of 965 normal hearts	P. T. Hagen	1984	New Engl J Med	200	8,774
6	Long-Term Outcomes of Patent Foramen Ovale Closure or Medical Therapy after Stroke	Jeffrey L Saver	2017	New Engl J Med	253	8,711
7	Closure of patent foramen ovale versus medical therapy after cryptogenic stroke	John D. Carroll	2013	New Engl J Med	257	8,634
8	Recurrent cerebrovascular events associated with patent foramen ovale, atrial septal aneurysm, or both	J. L. Mas	2001	Mayo Clin Proc	228	8,352
9	Prevalence of patent foramen ovale in patients with stroke	P. Lechat	1988	New Engl J Med	243	7,642
10	Effect of medical treatment in stroke patients with patent foramen ovale: patent foramen ovale in Cryptogenic Stroke Study	Shunichi Homma	2002	Circulation	159	6,786

### 3.6 Journals and co-cited journals analysis

Table 3 lists the top 10 journals with the highest number of publications and citations in the field of PFO closure. The top three journals by publication volume are Catheterization and Cardiovascular Interventions (n = 110), Journal of Interventional Cardiology (n = 28), and Journal of the American College of Cardiology: Cardiovascular Interventions (JACC: Cardiovascular Interventions, n = 24). Among these, JACC: Cardiovascular Interventions has the highest impact factor (IF = 11.7), while Stroke has the highest average number of citations per article (42.38 citations).

**Table 3:** Top 10 related popular journals.

Rank	Journals	Publications	Citations	Average citations	IF and JCR division(2023)
1	Catheterization and Cardiovascular Interventions	110	1811	16.46	2.1Q3
2	Journal of Interventional Cardiology	28	318	11.36	1.6Q3
3	JACC Cardiovascular Interventions	24	972	40.5	11.7Q1
4	Eurointervention	23	356	15.48	7.6Q1
5	American Journal of Cardiology	22	812	36.91	4.3Q1
6	International Journal of Cardiology	22	336	15.27	3.2Q2
7	Stroke	21	890	42.38	7.8Q1
8	Frontiers in Neurology	18	184	10.22	2.7Q2
9	Journal of Invasive Cardiology	18	96	5.33	1.6Q3
10	Echocardiography a Journal of Cardiovascular Ultrasound and Allied Techniques	16	205	12.81	1.7Q3

**Figure 6:**
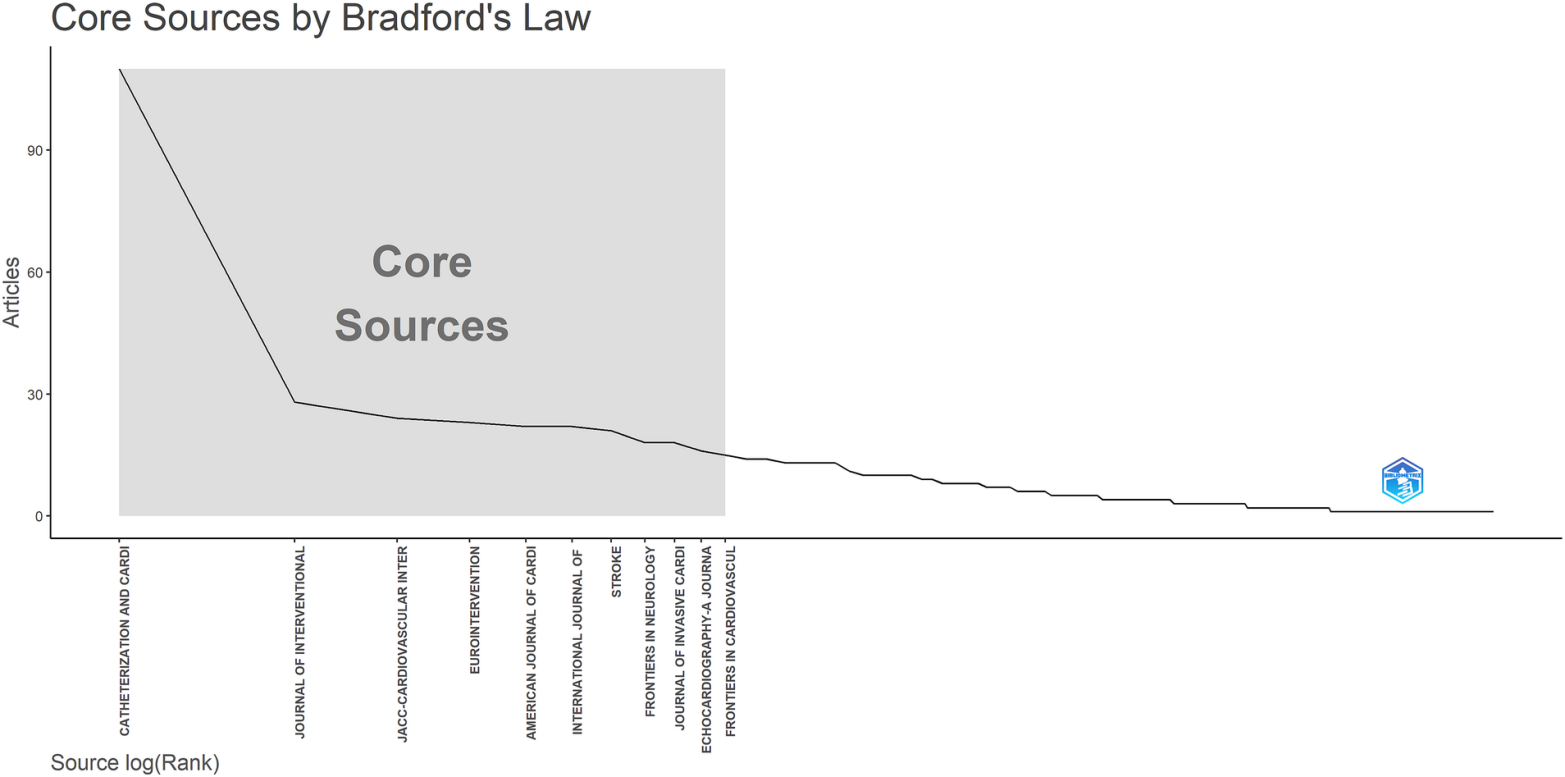
Core sources by Bradford’s law.

Core journals in this field were identified using Bradford’s Law and visualized with Bibliometrix R (Figure 6). The results fully align with those presented in Table 3. Bradford’s Law, also known as the law of scattering, describes the distribution pattern of scientific literature across journals, highlighting both the concentration and dispersion of research output as well as its zonal structure (10). Among the top 10 journals by publication volume, only Catheterization and Cardiovascular Interventions has published more than 100 articles, while the publication volume of other journals is less than 30 articles.

### 3.7 Keywords analysis

**Figure 7:**
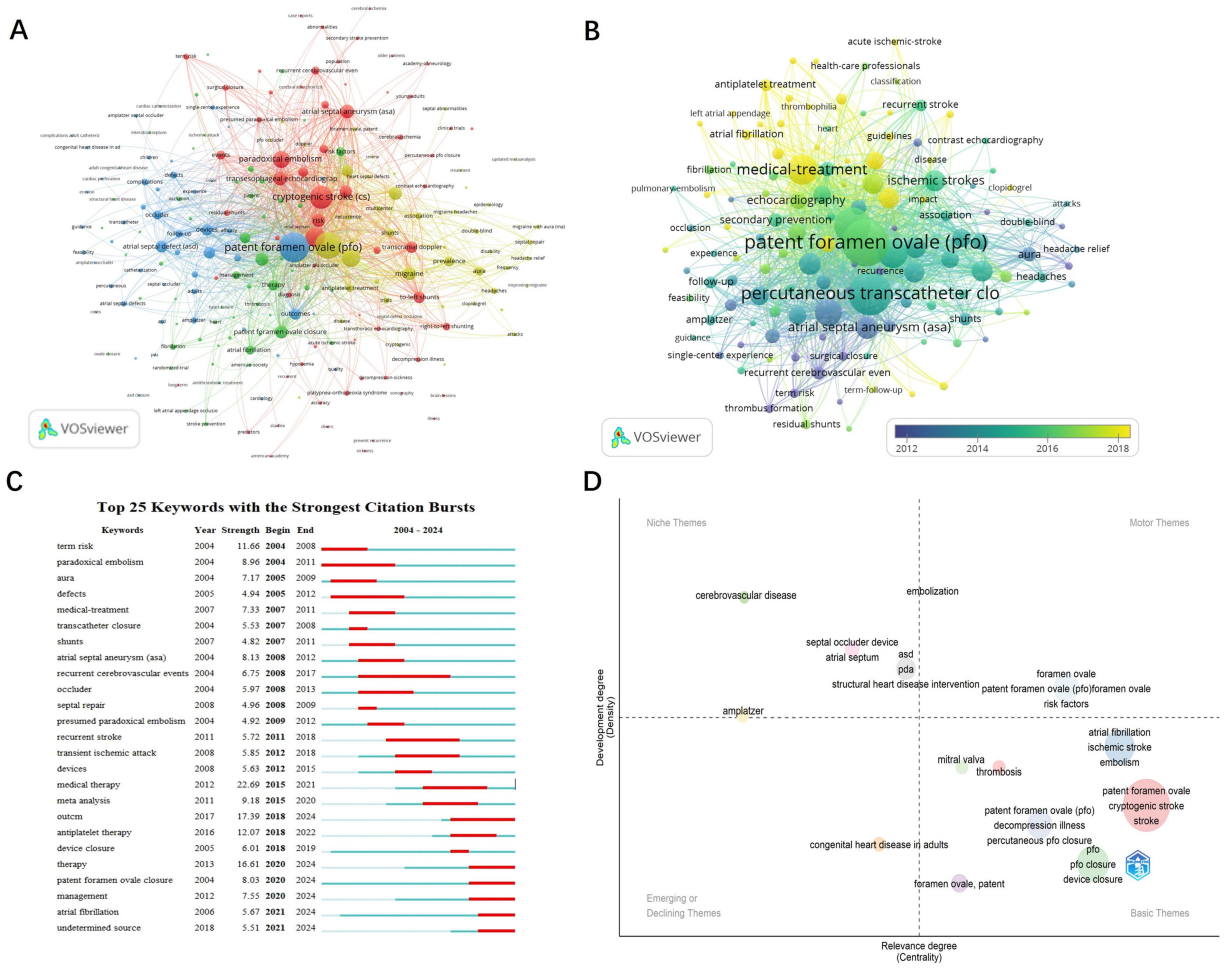
Keyword analysis in PFO closure research. **(A)** Visualization of keywords divided into 4 clusters. **(B)** Time overlay visualization of the keyword network. **(C)** The top 25 keywords with the strongest citation bursts. **(D)** Keyword trend topic map.

Keywords encapsulate the core themes of scientific publications and reflect the focal points of research in a given field. Through analyses of high-frequency keywords keyword co-occurrence and clustering scholars can gain insights into the current status development trends and research hotspots in the field of PFO closure. Additionally keywords with strong citation bursts can help identify emerging frontiers in the domain. Using VOSviewer, 1,671 keywords were analyzed, and 126 high-frequency terms (occurrence ≥10) were identified. After merging synonyms and excluding irrelevant terms, clustering analysis revealed four major research themes (Figure 7A). The red cluster centers on PFO-related clinical mechanisms, featuring keywords such as paradoxical embolism, stroke, and right-to-left shunting, underscoring the link between PFO and cryptogenic stroke. Diagnostic advancements, such as dynamic imaging during echocardiography, reflect a shift toward functional risk assessment. The blue cluster focuses on diagnostic techniques, including transesophageal echocardiography (TEE) and contrast echocardiography, which remain the gold standards for PFO detection. Innovations like 3D-TEE and bubble studies now allow for detailed assessment of high-risk anatomical features, aiding clinical decision-making regarding closure. The green cluster highlights treatment approaches, with prominent terms including PFO closure, percutaneous transcatheter closure, and Amplatzer devices. Since 2015, pivotal trials such as RESPECT and CLOSE have influenced a shift in treatment strategies, favoring minimally invasive closure over medical therapy for high-risk patients. The yellow cluster addresses outcomes and epidemiology, with terms such as recurrence, prevention, and follow-up, emphasizing the efficacy of PFO closure in reducing stroke recurrence. Emerging evidence also suggests secondary benefits, including migraine relief, further expanding the therapeutic value of the procedure. Together, these clusters outline the evolution of PFO closure research, from understanding pathophysiology to advancing diagnostics and implementing evidence-based interventions.

The temporal overlay visualization generated by VOSviewer illustrates the dynamic evolution of research priorities in PFO-associated pathologies, with acute ischemic stroke emerging as a central investigative focus (Figure 7B). It reveals three distinct phases: from 2012 to 2014, research concentrated on pathophysiology (e.g., paradoxical embolism, atrial septal aneurysm [ASA]) and diagnostic validation (e.g., contrast echocardiography), establishing PFO as a modifier of stroke risk. The period from 2015 to 2016 marked a rise in interventional strategies, characterized by the adoption of percutaneous closure techniques and Amplatzer occluders, reflecting increased clinical interest following device-based trial outcomes. From 2017 to 2018, the focus shifted toward optimizing outcomes, with studies addressing residual shunts, thrombus resolution, and migraine comorbidity. This shift illustrates the maturation of PFO management into a multidisciplinary paradigm integrating neurology, cardiology, and imaging. Notably, the post-2016 emergence of headache relief as a prominent term underscores growing recognition of migraine associated with PFO as a distinct therapeutic target.

Burst detection analysis (Figure 7C) further captures evolving trends in PFO management. Early bursts, such as paradoxical embolism (2004–2011) and risk (2004–2008), reflect initial efforts to establish a link between PFO and cryptogenic stroke. A decline in interest in transcatheter closure (2007–2008) followed inconclusive early trials (e.g., CLOSURE I), while renewed attention in 2018–2019 and a sustained focus on PFO closure from 2020 to 2024 coincided with key trial results (e.g., RESPECT Extended Follow-up, REDUCE), which supported closure in high-risk patients (e.g., those with large shunts or ASA). At the same time, medical therapy (2015–2021) remained relevant for low-risk populations, reflecting ongoing debates around the use of antiplatelet agents. More recent bursts, such as antiplatelet therapy (2018–2022) and management (2020–2024), highlight a growing interest in personalized treatment strategies, incorporating risk stratification tools (e.g., RoPE score) and shared decision-making frameworks. The emergence of atrial fibrillation (2021–2024) indicates heightened awareness of the interaction between PFO and AF, prompting a shift toward more refined diagnostic protocols, including extended cardiac monitoring to exclude occult AF prior to closure. Collectively, these shifts mark a transition from empirical treatments to evidence-based, patient-tailored strategies, driven by technological advancements (e.g., improved occluders) and evolving clinical guidelines, such as the 2020 AHA/ASA recommendations.

Figure 7D presents a thematic map of research trends in PFO studies, generated using Bibliometrix R. This analysis evaluates themes along two axes: centrality (horizontal), which reflects a theme’s relevance within the broader research network, and density (vertical), which indicates its level of conceptual development. Motor Themes (upper right quadrant), including patent foramen ovale, risk factors, and PFO closure, represent mature areas of the field, supported by landmark trials (e.g., RESPECT, CLOSE, DEFENSE-PFO) that established the clinical benefits of closure in specific subgroups (e.g., patients with large shunts or ASA). Niche Themes (upper left quadrant), such as Amplatzer devices, reflect technological innovation, exemplified by the FDA’s 2016 approval of the Amplatzer PFO Occluder. Basic Themes (lower right quadrant), including atrial fibrillation and ischemic stroke, highlight ongoing diagnostic challenges, particularly in differentiating PFO-related strokes from those caused by AF. Emerging or Declining Themes (lower left quadrant), such as adult congenital heart disease, indicate shifting research priorities influenced by clinical trial criteria (e.g., DEFENSE-PFO’s exclusion criteria). This thematic framework illustrates the process of knowledge translation: Motor Themes align with guideline-recommended practices, while Niche Themes represent technological innovation, together advancing the field toward precision medicine in PFO management.

## 4 Discussion

### 4.1 Clinical evolution of PFO closure

Research on PFO closure has undergone significant clinical evolution between 2004 and 2024. Early studies, such as those by Furlan et al. (2012) (11) and Meier et al. (2013) (12), reported inconclusive findings regarding the benefits of PFO closure over medical therapy for the prevention of cryptogenic stroke. These results initially dampened enthusiasm for closure as a treatment strategy. However, a landmark study by Mas et al. (2017) (13) published in the NEJM shifted the clinical paradigm by demonstrating that PFO closure combined with antiplatelet therapy significantly reduced the recurrence of cryptogenic stroke compared to antiplatelet therapy alone. This study reinvigorated interest in the field and contributed to a surge in research publications from 2018 to 2024.

### 4.2 Key clinical trials and their impact

Several pivotal clinical trials have helped establish the role of PFO closure in the secondary prevention of stroke. The RESPECT trial (2013) and its extended follow-up (2017) demonstrated that PFO closure significantly reduced the risk of recurrent stroke in patients with cryptogenic stroke. Similarly, the REDUCE trial (2017) confirmed the efficacy of closure in reducing stroke recurrence, particularly in high-risk patients. Collectively, these trials provide strong evidence supporting PFO closure as an effective intervention in carefully selected populations.

One particularly influential contribution to clinical practice has been the study by Kent et al. (14), which introduced the RoPE score. This tool helps differentiate between strokes that are likely attributable to a PFO and those in which the PFO may be incidental. The RoPE score aids clinicians in estimating the likelihood of stroke recurrence and in making informed decisions regarding PFO closure. Since its publication, this scoring system has been widely cited and has significantly influenced clinical guidelines and routine decision-making.

### 4.3 Contemporary clinical debates and unresolved issues

Our keyword burst analysis and thematic mapping highlight several ongoing clinical controversies that warrant further discussion: Upper Age Limit: Most randomized trials enrolled patients younger than 60 years; however, real-world registry data (Danish nationwide study (15)) suggest that PFO closure may also benefit older patients (aged 60–75) with high-risk anatomical or clinical features. Despite these findings, the optimal age threshold for closure remains a subject of debate (16). Residual Shunt: Residual shunts post-closure [observed in 5–25% of cases (17)] are associated with recurrent events (OR 3.1, 95% CI 1.4–6.9) (18). Advanced imaging (3D-TEE) and improved occluder designs (e.g., Amplatzer PFO Occluder) aim to mitigate this issue (19). TIA as a Qualifying Event: Current guidelines generally restrict PFO closure to patients with documented ischemic stroke, but emerging data [e.g., from the Gore REDUCE trial subgroup (20)] suggest potential benefit in high-risk TIA patients. Post-Closure Antiplatelet Duration: The 2020 AHA/ASA guidelines recommend 3–6 months of dual antiplatelets (21), yet recent studies propose individualized regimens based on thrombotic risk (22). Postprocedural AF Management: The Danish registry (15) reported a 7.8% 5-year AF risk post-closure, predominantly within 3 months. Early detection (e.g., with implantable loop recorders) and anticoagulation strategies are now prioritized (23).

## 5 Limitations

While this bibliometric analysis offers valuable insights into PFO-related stroke research, several methodological limitations should be acknowledged. First, the exclusive use of the Web of Science Core Collection (WoSCC) may introduce database bias, as WoSCC underrepresents non-English and region-specific journals (e.g., publications from China, Russia, or Latin America not indexed in this database). Although WoSCC was chosen for its rigorous journal selection criteria and compatibility with bibliometric tools such as VOSviewer, future studies could benefit from cross-validation using databases like Scopus or PubMed to address potential coverage gaps. Second, citation lag may affect the visibility of recent high-impact studies (2023–2024), as newer publications have not yet had sufficient time to accumulate citations, potentially underestimating emerging research trends. Third, author name disambiguation presents a challenge, particularly with common names (e.g., “Wang J.”), which may impact the accuracy of author collaboration networks and productivity metrics. Additionally, the exclusion of non-research articles (e.g., conference abstracts, letters) and non-English literature may limit the generalizability of findings, especially with respect to clinical practices in non-Western regions. These limitations highlight the need for cautious interpretation of bibliometric results and underscore the importance of broader, more inclusive analyses in future research.

## 6 Conclusion

As the first bibliometric study focused on PFO closure, this work provides a comprehensive and objective overview of the current landscape and clinical research trends in this field. With continued advances in our understanding of PFO-related diseases, the use of PFO closure as a therapeutic intervention is expected to become increasingly evidence-based, targeted, and effective in the future.

## Data Availability

The raw data supporting the conclusions of this article will be made available by the authors, without undue reservation.

## Footnotes

^1^ https://bibliometric.com/
